# Managing Conflict Case: Admission of a Patient with Decompensated Schizophrenia, Hypertension, and Diabetes

**DOI:** 10.21980/J8.52341

**Published:** 2025-12-31

**Authors:** Monique Graf, Sonika Raj, Jedidiah Leaf, Marshall Howell, Joshua Ginsburg, Brian Milman, Samuel Parnell

**Affiliations:** * Baylor Scott & White All Saints Medical Center-Fort Worth, Department of Emergency Medicine, Fort Worth, TX; ^ University of Texas Southwestern Medical Center, Department of Emergency Medicine, Dallas, TX

## Abstract

**Audience:**

This practice Certifying Exam Communication case is intended for emergency medicine resident physicians (both junior and senior).

**Introduction:**

Conflict is the result of two parties who differ in their expectations, agendas, personal needs, backgrounds, and/or communication styles. Emergency medicine physicians are faced with conflict in various forms in the workplace on a regular basis related to task content, interpersonal differences, and processes.^1^,^2^ When conflict is not managed appropriately, it can result in subpar patient care and poor team morale.^3^,^4^ Thus, conflict management is an important skill for all emergency medicine physicians. With this in mind, ABEM (the American Board of Emergency Medicine) has now chosen to assess candidates’ conflict management proficiency as part of its new Certifying Exam.^5^ As educators, we must prepare trainees for both the certification exam and the clinical setting by creating opportunities for practice, focused evaluation, and formative feedback. To strengthen our certifying exam preparation curriculum, we designed a certifying exam practice case to help residents learn how to manage conflict.

**Educational Objectives:**

The following objectives, which align with ABEM’s recommended objectives, address the nuances of managing conflict. By the end of the session, learners should be able to: 1) demonstrate familiarity with the ABEM Managing Conflict case format and structure, 2) establish rapport by developing connection and trust with the admitting physicians, 3) demonstrate understanding of the other party’s position by verbalizing thoughtful and specific questions about his/her concerns, 4) explain his/her own position clearly and insightfully, 5) acknowledge divergent positions with thoughtfulness and insight, 6) identify interests shared by both the psychiatrist and hospitalist as well as what is in the best interest of the patient, and 7) propose a path forward which accounts for the interests of all parties involved.

**Educational Methods:**

We developed a 10-minute OSCE (Objective Structured Clinical Examination)-style case requiring the resident examinee to manage a conflict with consulting physicians. The ABEM certifying exam will utilize standardized patient actors. For our case, the faculty examiner served as the actor who alternated between the two roles required by the case.

**Research Methods:**

We piloted this case with 18 PGY-3 emergency medicine residents. We developed a grading rubric based on ABEM’s six published conflict-management case learning objectives. To pass the case, learners had to score 12 or more of the 18 available points (67%). Each learner was also asked to complete a post-case evaluation associated with a 5-point Likert scale.

**Results:**

When asked whether the case increased their understanding of the new ABEM certifying exam format, 18/18 (100%) of the participants replied “agree” or “strongly agree." When asked to assess the overall quality of the case, 16/18 (88.89%) of the participants stated that it was “very good” or “excellent.” The case received a mean score of 4.61/5. The mean critical action completion score was 17.61/18.

**Discussion:**

We sought to create a practice certifying exam case that provided learners with a high-fidelity opportunity to both understand ABEM’s new certifying exam requirements and to be evaluated on how well they managed conflict. Our results reflect that most learners felt we addressed these aims, and that overall, our educational assessment was effective.

**Topics:**

Conflict management, communication, certifying exam.

## USER GUIDE

**Table:** 

List of Resources:	
Abstract	30
User Guide	32
For Examiner Only	34
Certifying Exam Assessment	38
Stimulus	39
Debriefing and Evaluation Pearls	41

**Learner Audience:**

This exercise is appropriate for medical students, interns, and junior and senior residents.

**Time Required for Implementation:**

Case: 10 minutes

Debriefing: 5 minutes

**Recommended number of learners per instructor:**

one

**Topics:**

Conflict management, communication, certifying exam

**Objectives:**

By the end of this session, the learner will be able to:

1. Demonstrate familiarity with the ABEM Managing Conflict case format and structure.
2. Establish rapport by developing connection and trust with the admitting physicians.
3. Demonstrate understanding of the other party’s position by verbalizing thoughtful and specific questions about his/her concerns.
4. Explain his/her own position clearly and insightfully.
5. Acknowledge divergent positions with thoughtfulness and insight.
6. Identify interests shared by both the psychiatrist and hospitalist as well as what is in the best interest of the patient.
7. Propose a path forward which accounts for the interests of all parties involved.

### Linked objectives, methods and results

These objectives are achieved by the OSCE format in that a communication-based skill such as conflict management requires interpersonal interactions.

Implementation of this conflict management simulation into our curriculum allows residents to practice and reflect upon the skill of conflict management in alignment with Kolb’s experiential learning cycle.^6^ Kolb’s experiential learning cycle theory is based upon the idea that learning is not linear, but rather a continuous, four-stage cycle of concrete experience, reflective observation, abstract conceptualization, and active experimentation.^10^ It has been established that residents have concrete experience with conflict management on shift. However, given the fast-paced demands of the emergency department, there is limited time for reflective observation. Our simulation allows residents to take their learning a step further from just experience. They can reflect on the case and utilize abstract conceptualization via debriefing sessions and discussions about conflict management strategies taught and utilized by experienced attending physicians. Our residents then have the opportunity to experiment in the clinical setting with new conflict management strategies, thereby paving the way for them to learn what works best for them to achieve high-quality patient care.

### Recommended pre-reading for instructor

- American Board of Emergency Medicine. *Certifying Exam Case Materials: Managing Conflict.* American Board of Emergency Medicine; 2024. Accessed November 19, 2025. https://www.abem.org/wp-content/uploads/2024/11/Case-Materials_Managing-Conflict.pdf
- American Board of Emergency Medicine. *Certifying Exam Scoring Information*. American Board of Emergency Medicine; 2024. Accessed November 19, 2025. https://www.abem.org/wp-content/uploads/2024/11/ce-scoring-information.pdf
- Chan T, Bakewell F, Orlich D, Sherbino J. Conflict prevention, conflict mitigation, and manifestations of conflict during emergency department consultations. *Acad Emerg Med.* 2014;21(3):308–313. Accessed 12/5/2025. https://doi:10.1111/acem.12325
- O’Mara K. Communication and conflict resolution in emergency medicine. *Emerg Med Clin North Am*. 1999;17(2):451–xii. Accessed 12/5/2025. doi:10.1016/s0733-8627(05)70071-7
- Tjan TE, Wong LY, Rixon A. Conflict in emergency medicine: a systematic review. *Acad Emerg Med*. 2024;31(6):538–546. Accessed 12/5/2025. doi:10.1111/acem.14874

### Results and tips for successful implementation

Results:

We piloted this case with 18 PGY-3 emergency medicine residents. We developed a grading rubric based on ABEM’s six published conflict management case learning objectives. To pass the case, learners had to score 12 or more of the 18 available points (67%). Each learner was also asked to complete a post-case evaluation consisting of two items, each associated with a 5-point Likert scale:

1. This case increased my understanding of the certifying exam format: Answer options: Strongly Disagree, Disagree, Neutral, Agree, Strongly Agree
2. How would you rate the overall quality of this case? Answer options: Poor, Fair, Good, Very Good, Excellent

When asked whether the case improved their understanding of the new ABEM certifying exam format, 18/18 (100%) of the participants replied “agree” or “strongly agree." When asked to assess the overall quality of the case, 16/18 (88.89%) of the participants stated that it was “very good” or “excellent,” and the case received a mean score of 4.61/5. The mean critical action completion score was 17.61/18. The lowest-scoring categories were “understand the other person’s position” and “identify shared interests,” while the highest-scoring category was “explain his/her own position.”

After all our learners completed the case, a group debrief was held to reinforce teaching points and to elicit group feedback on the case. During this debrief, many learners expressed that this case felt like real-life circumstances and thus provided a generalizable framework that could be applied to the clinical setting. This sentiment led to discussions about structured approaches to managing conflict in the emergency department.

We administered this case with one standardized actor (the faculty examiner) who played both psychiatrist and hospitalist. While this format was chosen based on the number of available examiners, it could be improved by employing two standardized actors – one to play each clinician – or even one standardized actor who is not the examiner. Either of these modifications would likely enhance the fidelity of the scenario.

One key lesson we learned from running this case was the importance of adhering to the outlined timing for each sub-discussion and utilizing the included prompts as needed. However, the most important takeaway from implementing this case was how much learners valued being able to learn conflict management and receive individualized feedback. The overwhelming sentiment among our learners was that understanding how to manage conflict well is essential to the provision of high-quality patient care. Therefore, we believe that educators must continue to seek opportunities to help learners develop and strengthen the skill of managing conflict.

Tips for successful implementation:

1. We administered this case with one standardized actor (the faculty examiner) who played both psychiatrist and hospitalist. This format could be modified by employing two standardized actors – one to play each clinician – or even one standardized actor who is not the examiner. Any modification in the number of standardized actors would require attention to case timing, i.e., allowing time for the actors to enter and leave the room. In all cases, it remains important to adhere to the recommended timings to ensure case completion. It is also critical that at the start of each sub-discussion, the standardized actor introduces and re-introduces himself/herself by name and title in order to avoid confusing the examinee.
2. We utilized a passing score of 12/18 and noted that most learners scored well above that. In future iterations of this case, one might consider a higher passing score in order to uncover more subtle differences in learners’ strengths and weaknesses.

## Figures and Tables

**Table:** Managing Conflict Candidate Task Sheet

**CASE PARAMETERS**	
This is a 10-minute case. You will interact with a psychiatrist and a hospitalist regarding a 40-year-old male with schizophrenia who presented to the ED with hallucinations and delusions despite taking his prescribed medication. Overnight, the patient received medications which improved his symptoms. You recommend admission for psychiatric medication adjustment and monitoring, and paged the psychiatrist to the ED. The psychiatrist just arrived. Note: per protocol, the psychiatry unit may give home medications.	
**PATIENT INFORMATION**	
**Patient Name**	Mr. John
**Age**	40
**Gender Identity**	Male
**Presenting complaint**	Hallucinations and delusions
**General appearance/History of present illness**	General appearance: calm and cooperative History of present illness: initially presented with hallucinations and delusions despite taking his prescribed medication
**Past Medical History**	Schizophrenia, diabetes, hypertension
**Medication**	Ziprasidone, insulin, amlodipine
**Allergies**	None
**Vital Signs on ED arrival**	T 98.6º F, BP 165/97, HR 78, RR 16, SpO2 100% on room air
**RESULTS/FINDINGS**	
ED course: Overnight, the patient was severely agitated. Medical management (50 mg Benadryl, 2 mg Ativan, and 5 mg Haldol) improved his symptoms, and he is now calm and cooperative. Physical exam: normal Labs: BMP is normal except for glucose of 235 mg/dL. All other diagnostic tests, including the urine drug screen, are normal.	
**TASK STATEMENT**	
Your task is to speak with both the psychiatrist and the hospitalist to facilitate admission.	

## CERTIFYING EXAM ASSESSMENT

*Admission of a Patient with Decompensated Schizophrenia, Hypertension and Diabetes*

Learner: _________________________________________

**Establish rapport**

□ Introduces self.
□ Develop a connection and trust with both admitting physicians, which, despite the conflict at hand, creates a positive environment.

**Understand the other person’s position**

□ Identifies more than one thoughtful and specific question about the other physicians’ concerns.
□ Remains respectful and non-confrontational.

**Learners explain their own position**

□ Is concise.
□ Communicates rationale clearly and insightfully.

**Acknowledge divergent positions**

□ Verbalizes understanding of differing opinions.

**Identify shared interests**

□ Redirects conversation toward shared goals by identifying more than one shared interest between the psychiatrist and hospitalist as well as what is in the best interest of the patient.
□ Frames the discussion around what is best for the patient.

**Propose a path forward**

□ Summarizes the agreed-upon plan which accounts for the interests of all parties involved.
□ Confirms mutual understanding.

**Summative and formative comments:**

### Stimulus Inventory

**Candidate Task Sheet**

## DEBRIEFING AND EVALUATION PEARLS

### Admission of a Patient with Decompensated Schizophrenia, Hypertension, and Diabetes

What is conflict?

Conflict is the result of two parties who differ in their expectations, agendas, personal needs, backgrounds, and/or communication styles.

What are the tenets of conflict resolution?

Conflict resolution requires that you:

- Ensure appropriate timing and a private, distraction-free environment in which to have this discussion
- Describe the conflict in an objective, fact-based manner
- Establish rapport by developing a strong connection and trust with the other party, which despite the conflict at hand, creates a positive environment
- Understand the other party’s position by asking thoughtful questions, listening actively, and acknowledging divergent positions with empathy
- Explain your own position
- Depersonalize the problem to avoid being perceived as judgmental
- Identify interests and desired outcomes shared by all parties involved
- Negotiate a path forward which accounts for the interests of all parties involved

Do we have any evidence-based approaches to conflict management?

The DESC model, developed by psychologist Sharon Bower and communication expert Gorder Bower, is designed to minimize defensiveness while preserving one’s ability to be assertive.^7^ It is best used in cases of interpersonal conflict and especially appropriate when a patient’s or team member’s physical or emotional safety is at risk. The four steps of the DESC model are Describe, Express, Suggest, and Consequences. First, the negotiating party describes the issue at hand, while taking care to remain objective and fact-based. Next is an expression of how the situation makes the first party feel. Using “I” statements in this step can be helpful in warding off a defensive response. Next, one suggests the desired outcome as well as any acceptable alternatives. Lastly, one states the consequences of that desired outcome as well as the consequences of that outcome not happening.

When using this tool, it is important to consider the timing and location of the discussion. Appropriate timing and a private, distraction-free environment allow both parties to focus on the issue itself rather than being right or avoiding embarrassment.

Suggested questions to guide the debriefing session

- How did you feel about your performance during this case?
- What about this conflict felt manageable, and why? What felt challenging, and why?
- How well do you feel you met the above-mentioned tenets of conflict resolution?
